# Food supplementation with wheat gluten leads to climbing performance decline in *Drosophila melanogaster*

**DOI:** 10.17912/micropub.biology.000642

**Published:** 2022-09-23

**Authors:** Naphtali Qely Remy, Justine Anne Guevarra, Fernando J Vonhoff

**Affiliations:** 1 University of Maryland Baltimore County, Baltimore, MD, United States

## Abstract

Gluten sensitivity is associated with digestive and neurological disorders, correlating with abnormal amino acid levels, innate immune responses, gut dysbiosis and movement incoordination. However, the molecular mechanisms linking dietary gluten and brain function remain incompletely understood. We used *Drosophila melanogaster* to test the effects of gluten ingestion in locomotion performance. Whereas flies on control food showed decreased climbing performance after five weeks, flies exposed to food supplemented with different gluten concentrations showed a significant locomotion decline after three weeks of treatment. Future studies will determine the mechanisms underlying the observed gluten-dependent phenotypes to establish *Drosophila* models for gluten sensitivity.

**Figure 1. An accelerated decline in climbing performance is observed in flies exposed to food supplemented with gluten:**
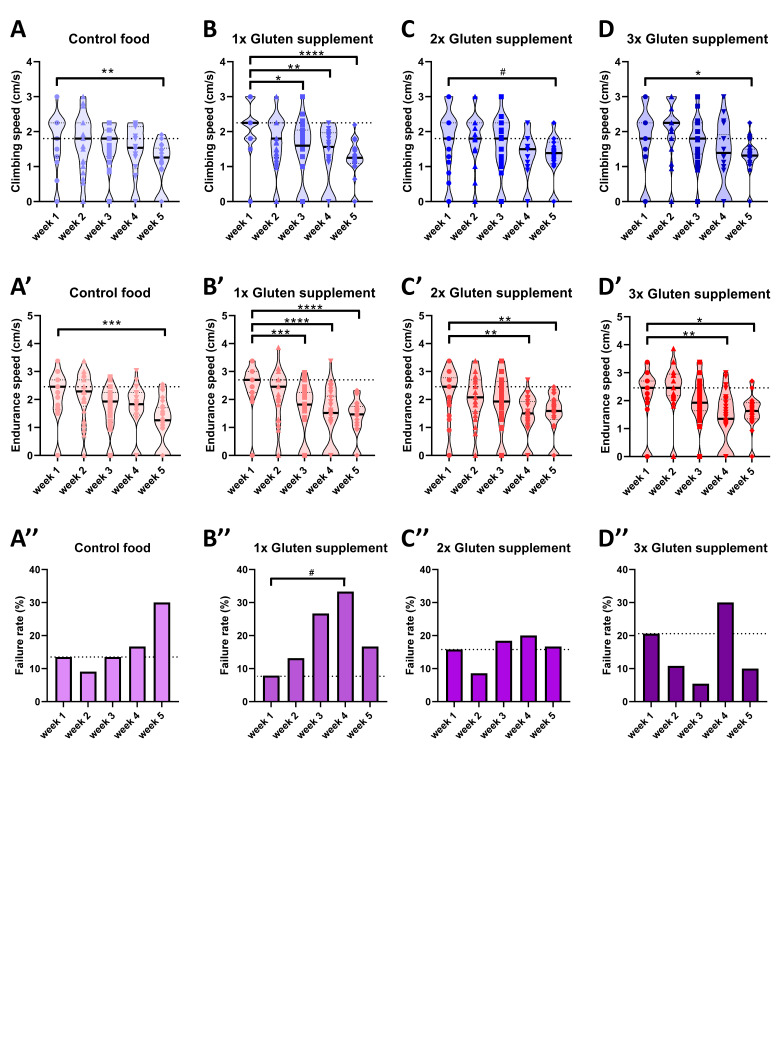
Climbing and endurance speed was calculated in wildtype flies raised on control food (A) or on food supplemented with 1x (B), 2x (C), or 3x (D) gluten after one to five weeks of food treatment. These datasets are also presented in an additional way as a table in the Extended Data section of the manuscript. Violin plots show the distribution of all measured data points in aligned form, representing the median and the quartiles with a solid or a dotted line inside of the plot, respectively. The large, dotted lines represent the median value of performance of flies after one week of treatment. The non-parametric Kruskal-Wallis one-way ANOVA with Dunn’s multiple comparisons test was used for climbing and endurance speed, and the Fisher’s Exact Test and chi square analysis were used for failure rate analysis. *P < 0.05, **P < 0.01, ***P < 0.001, ****P < 0.0001, #P = 0.06.

## Description

In recent decades, studies have focused on how wheat consumption has contributed to the spike in several illnesses such as celiac disease, irritable bowel syndrome, and neuro-psychiatric disorders (Sapone et al., 2012; Kasarda, 2013; Catassi, 2015; Biesiekierski and Staudacher, 2020). The main structural constituent in wheat and other grass-related grains is gluten, which is a complex mixture of hundreds of distinct, but related protein components (including gliadins and glutenins) and some lipids (Wieser, 2007; Biesiekierski, 2017). In susceptible individuals, exposure to dietary gluten may lead to allergic (as in wheat allergy) or autoimmune reactions (e. g. celiac disease), as well as non-celiac gluten sensitivity (NCGS), which refers to immune reactions leading to gluten-induced symptoms but in the absence of autoantibodies (Biesiekierski et al., 2011; Sapone et al., 2012; Dale et al., 2019; Cárdenas-Torres et al., 2021). Furthermore, in some gluten intolerant patients, ingestion of gluten has also been associated with neurological disorders such as schizophrenia, autism, depression, and ataxia (Catassi, 2015; Leffler et al., 2015; Levinta et al., 2018; Aranburu et al., 2021). Gluten ataxia refers to idiopathic cases of movement disorders in patients with immune responses to gluten (Briani et al., 2008; Khwaja et al., 2015), whose symptoms can improve following gluten-free diets (Mearns et al., 2019; Patel et al., 2021). Intriguingly, the gut microbiome has been implicated in the pathogenesis of gluten-dependent celiac disease and psychotic symptoms either via the “leaky gut” hypothesis (abnormal intestinal permeability) (Camilleri, 2021) or via innate immune responses (Lionetti et al., 2015; Uche-Anya and Lebwohl, 2021). Additionally, gluten ingestion has also been described to lower the ratio of plasma tryptophan to other large neutral amino acids in rats (Choi et al., 2009) and humans (Fernstrom et al., 2013), affecting brain tryptophan uptake and serotonin synthesis. By contrast, a different study reported elevated levels of tryptophan in plasma samples from children with celiac disease (Torinsson Naluai et al., 2018), supporting the possible role of abnormal amino acid metabolism and nutrient sensing signaling pathways in disease pathogenesis. However, the molecular mechanisms regulating the interactions between gluten ingestion and brain function remain incompletely understood.

*Drosophila* has served as a powerful model in food and nutrition research involving conserved molecular mechanisms of nutrient sensing pathways. For example, recent studies have focused on the regulation of internal states and behavioral decision making (Anderson, 2016; Khan et al., 2021; Vogt et al., 2021), and, at the molecular level, how fly adipocytes respond to dietary changes and regulate stem cell lineages (Armstrong, 2020). Also, the relatively simple *Drosophila* gut microbiota has been used as a model to study mammalian gut complexity and microbiome/drug interactions (Douglas, 2018; Ludington and Ja, 2020), as well as its role in fly disease models such as autism (Salim et al., 2021) or Alzheimer’s disease (Jalali et al., 2021; Kitani-Morii et al., 2021). However, whether gluten ingestion affects neuronal function and behavioral performance in *Drosophila* remains a largely unexplored question. Here, we used *Drosophila melanogaster* to test the effects of dietary gluten on locomotor performance.

We used a previously described method to calculate the speed of wildtype flies traveling a short distance (climbing speed) and a long distance (endurance speed) after exposure to different food treatments (Gabrawy et al., 2019; Gabrawy et al., 2022). Flies were fed different concentrations of wheat gluten over five weeks and their behavioral performance was analyzed at initial and late time points of gluten consumption. Consistent with previously published data (Gabrawy et al., 2019; Gabrawy et al., 2022), we observed flies on control food to show a significant decrease in climbing and endurance speed after five weeks (Fig. 1A, A’). Interestingly, flies on gluten-supplemented food showed a significant decline in climbing and endurance speed as early as three weeks of treatment (as in the case of supplementation of 1x gluten; Fig. 1B, B’). Interestingly, our data indicate that 1x gluten supplementation had a stronger effect on climbing and endurance speed than higher gluten concentrations. Whereas experimental evidence to explain this observation is still lacking, one parsimonious explanation involves the possibility that flies exposed to high levels of gluten were able to properly activate homeostatic mechanisms already after the initial days of exposure. By contrast, 1x gluten supplementation might not have been enough to trigger such compensatory responses during the first weeks of exposure, which, combined with aging-dependent factors, significantly affected the fly’s behavioral performance after 3 weeks of gluten exposure. Future studies would be necessary to assess age-dependent effects of gluten supplementation in young and old flies.

Furthermore, it is worth noting that the effect of gluten supplement was stronger on endurance speed than climbing speed. This effect is not only observed in more drastic decreases for endurance speed than climbing speed within the 1x gluten group, but also by the fact that a significant decline in endurance speed is observed at week 4 and week 5 for the 2x and 3x groups as compared to a relatively significant effect on climbing speed only until week 5. A parsimonious explanation for this effect involves the possibility that the motor network is challenged at a higher level becoming more susceptible to show fatigue-associated effects when a long distance is travelled for the measurement of endurance speed as compared to a three-fold shorter distance travelled for climbing speed. The rationale for this idea is based on differences in physiology between muscle types (Schiaffino and Reggiani, 2011; Spletter and Schnorrer, 2014). Leg muscles in *Drosophila* are mostly glycolytic, which are closely related to fast-twitch muscles in mammals and fatigue rather quickly (Röckl et al., 2007; Piccirillo et al., 2014; Damschroder et al., 2020). By contrast, *Drosophila* flight muscles are mostly oxidative muscles, closely related to mammalian slow-twitch muscles, and are more fatigue-resistant (Röckl et al., 2007; Piccirillo et al., 2014; Damschroder et al., 2020). Therefore, small differences in motor performance will be more easily distinguishable through analysis of endurance than climbing.

The gluten-dependent decline in locomotion performance (climbing and endurance) observed in this study could not be explained by an increase in failure rate (defined as the percentage of flies unable to reach the 9 cm mark; Fig. 1 A”-D”), as this measurement was not significantly different between controls and any of the gluten groups. It is worth mentioning that the failure rate for the 1x gluten group at week 4 (33.33%) is more than three-fold higher than the failure rate at week 1 (7.9%). This difference would have been statistically significant as using a two-sided Fischer’s exact test results in a p= 0.0122. However, despite its precise nature, Fischer’s exact test is less suitable for testing more than two groups, in which case a chi-square test is more recommended and used as default by most statistics packages (Kim, 2017). Therefore, we analyzed our data using a chi-square test due to the inclusion of weeks 1-5, resulting in a p=0.0550 for the 2x gluten supplementation group. Overall, our results suggest that weeks-long gluten exposure might have a negative effect on locomotor performance in flies.

Although our data suggest some resemblance to the movement defects observed in gluten ataxia patients as described above, these results should be interpreted with caution due to some limitations of the fly model and the preliminary nature of the data. Gluten ataxia is usually associated with cerebellar damages (Briani et al., 2005) but the adult fly brain lacks a cerebellum (Scheffer et al., 2020). However, the possibility that gluten-dependent effects might act on discrete regions of the fly central nervous system that control movement and coordination (Akitake et al., 2015; DeAngelis et al., 2019) cannot be ruled out, as previous studies have shown changes in locomotor behavior following misregulation of the motor network including motoneurons (Duch et al., 2008; Vonhoff et al., 2012; Mishra-Gorur et al., 2014; Ryglewski et al., 2014; Ryglewski et al., 2017). Also, it remains unknown whether ingestion of gluten would lead to similar reactions in the digestive system in flies and mammals. In humans, gluten interactions with the Transglutaminase 2 (TG2) enzyme are thought to be involved in the immunopathogenesis of celiac disease via the formation of TG2-gluten complexes and TG2-catalyzed deamidation of gluten peptides (Klöck et al., 2012; Amundsen et al., 2022; Paolella et al., 2022). A TG2 homolog with numerous conserved molecular properties is present in the fly genome (Iklé et al., 2008) and its inhibition attenuated neurodegeneration in a fly model of Huntington’s Disease (McConoughey et al., 2010). Future studies would be required to confirm the level of conservation between the two systems.

Despite such limitations, most of the multiple effectors of gluten described above are conserved in flies, such as gut microbiota (Arias-Rojas and Iatsenko, 2022) or immune responses (Campbell et al., 2020; Kietz and Meinander, 2022), whose modulation can lead to behavioral changes. In fact, effects on fly locomotion following gut dysbiosis have been previously reported, such as hyperactive (Schretter et al., 2018) and moderately elevated (Selkrig et al., 2018) locomotor behavior in germ-free flies. By contrast, flies fed with food supplemented with chloroform/methanol-soluble protein 3 (CM3), the most abundant amylase trypsin inhibitor in wheat, showed increased intestinal bacterial load and decreased lifespan, but no effect on climbing performance (Thiel et al., 2020). One hypothesis would include the possibility that ingestion of gluten in adult flies affects intestinal size (as previously reported for larvae) (Keelani, 2018) as well as the fly gut microbiome, which in turn would affect locomotion performance. A possible mediator in this scenario would involve the fly innate immune response, as the absence of gut microbiota in flies reduces age-dependent activation of innate immunity genes (Shukla et al., 2021), which are known to regulate neurodegeneration (Shukla et al., 2019). Accordingly, climbing performance may be negatively affected by changes in innate immune responses following gluten-dependent gut dysbiosis. An alternative hypothesis involves a possible gluten-dependent change in serotonin levels, as disruption of the serotonergic system has been previously described to significantly affect locomotion (Majeed et al., 2016; Howard et al., 2019). Future studies would address the intriguing question of whether (and how) gluten ingestion in flies would affect the gut microbiome, serotonin levels, innate immune responses, or degeneration of the motor network.

## Methods

We used wildtype males of the line DGRP-774 (RRID:BDSC_25205) from the Drosophila Genome Reference Panel lines, which are fully sequenced inbred stocks derived from a natural population from Raleigh, NC (Mackay et al., 2012). Females were excluded in this study to avoid additional effects on fertility, egg production, and lifespan. The DGRP-774 fly stock was obtained from the Bloomington Drosophila Stock Center (BDSC) and has previously served as a useful wildtype fly line showing sexually dimorphic ethanol-dependent behaviors (Oyeyinka et al., 2022). Flies were kept at 25°C in a 12/12 h light/dark incubator on standard plastic vials. Flies were cultured on a regular cornmeal/molasses food as described before (Oyeyinka et al., 2022). We used gluten from wheat (Sigma G5004) in powder form, which was added to our standard food as supplement in different concentrations. The amount of gluten added as a supplement was based on the set amount of protein estimated in each vial for our standard control food, considering the >75% amount of protein present in the gluten powder. For the 1x gluten supplement, 0.1434 grams of wheat gluten was added to each vial containing 10 ml of standard food, whereas 0.2868 g and 0.4302 g of gluten were added for the 2x and 3x supplementation, respectively. Adult flies were collected after eclosion on vials with regular food for 1-3 days until there were enough flies to start experiments for all gluten concentrations at the same time. Flies were transferred to regular and gluten-containing food on the same day, with fresh food vials being replaced every 3-4 days.

We used a previously described locomotion assay to examine the effects of gluten ingestion on fly climbing behavior (modified from (Gabrawy et al., 2019; Gabrawy et al., 2022)). Briefly, using 5ml volumetric pipettes, a mark was placed 9 cm from the 0 ml mark, which was used to determine climbing speed (in cm/s) by measuring the time each fly needed to travel such short distance. A second mark was placed 27 cm from the 0 ml mark to measure the time for a fly to travel such long distance, defined as endurance speed (in cm/s). The timer began once the fly started from the 0 ml mark on the bottom of the pipette and stopped once the fly crossed the 27 cm mark on the top. During this time, the climbing speed was recorded at the time the fly crossed the first 9 cm mark. 10 wildtype fruit flies per gluten concentration were tested on a weekly basis until 5 total weeks were recorded and repeated until a sample size of at least n= 30 flies for each condition was obtained. On a testing day, each fly was tested once, which was then recovered, placed back in the corresponding food vial, tested again the following week, and repeated until data for week 5 after exposure was collected. Climbing failure rate was calculated as the number of flies unable to complete the 9 cm distance due to stopping or falling.

**Statistical Analysis** :

Graphs and statistical analysis were performed using GraphPad Prism 9.4.1.681. Normality was tested using the Shapiro–Wilk test, with most groups not showing a normal distribution. Statistical significance was established using the non-parametric Kruskal-Wallis one-way ANOVA with Dunn’s multiple comparisons test for climbing and endurance speed, whereas the Fisher’s Exact Test and Chi Square analysis were used for failure rate analysis. We used flybase.org as an essential database for our studies (Larkin et al., 2021).

## Reagents

Wildtype flies from the stock DGRP-774 (Bloomington stock line BDSC_25205) were raised on standard control food or food supplemented with different concentrations of wheat gluten (Sigma G5004, CAS number: 8002-80-0).
